# Examining efficacy and safety of ethyl acetate extract from *Allium hirtifolium* as complementary therapy in COVID-19: A randomized, multicenter, controlled clinical trial

**DOI:** 10.22038/AJP.2024.24523

**Published:** 2024

**Authors:** Mansour Amin, Mandana Pouladzadeh, Mohammad Jaafar Yadyad, Roshanak Roshanfard, Mohamad Hasan Pipelzadeh, Afshin Talebi, Behzad Sharif Maakhmalzadeh, Mehdi Bijanzadeh, Nasrin Rakipour, Gholamreza Alizadehattar, Seyed Saeed Seyedian, Kambiz Ahmadi Angali, Parastoo Moradi Choghakabodi, Akbar Akbari, Fatemeh Maghsodi, Ebrahim Barzegari, Maryam Jamalan, Reza Aalizadeh, Mostafa Jamalan

**Affiliations:** 1 *Department of Microbiology, Infection and Tropical Diseases Research Center, School of Medicine, Ahvaz Jundishapur University of Medical Sciences, Ahvaz, Iran*; 2 *Department of Emergency Medicine, School of Medicine, Ahvaz Jundishapur University of Medical Sciences, Ahvaz, Iran*; 3 *Department of Infectious Diseases, School of Medicine, Ahvaz Jundishapur University of Medical Sciences, Ahvaz, Iran*; 4 *Department of Pharmacology and Toxicology Research Centre, Ahvaz Jundishapur University of Medical Sciences, Ahvaz, Iran*; 5 *Department of Internal Medicine, School of Medicine, Razi Hospital, Ahvaz Jundishapur University of Medical Sciences, Ahvaz, Iran*; 6 *Nanotechnology Research Center, School of Pharmacy, Ahvaz Jundishapur University of Medical Sciences, Ahvaz, Iran*; 7 *Department of Medical Genetics, School of Medicine, Ahvaz Jundishapur University of Medical Sciences, Ahvaz, Iran*; 8 *Department of Biostatistics and Epidemiology, School of Health, Ahvaz Jundishapur University of Medical Sciences, Ahvaz, Iran*; 9 *Department of Biochemistry, Faculty of Medicine, Abadan University of Medical Sciences, Abadan, Iran*; 10 *Medical Biology Research Center, Health Technology Institute, Kermanshah University of Medical Sciences, Kermanshah, Iran*; 11 *Department of Microbiology and Immunology, School of Medicine, Kashan University of Medical Sciences, Kashan, Iran*; 12 *Department of Biochemistry, Faculty of Biological Science, Tarbiat Modares University, Tehran, Iran*

**Keywords:** SARS-CoV-2, COVID-19, Lopinavir, Ritonavir, Shallomin syrup, Drug safety

## Abstract

**Objective:**

Given the apparent life-threatening nature of COVID-19, finding an effective treatment is under investigation.

**Materials and Methods:**

We assessed effect of shallomin oral syrup (co IranAmin^®^) as a complementary treatment to improve the clinical outcomes in COVID-19 patients. Patients in the control group received the approved treatment protocol (lopinavir/ritonavir), while those in the intervention group were treated with the oral syrup shallomin in addition to the approved treatment. Clinical status of treated patients was recorded and compared.

**Results:**

There were meaningful differences between the two groups regarding shortened length of hospital stay and the recovery time for cough, myalgia, sore throat, and shortness of breath. No side effect occurred in the intervention group compared to the control group in terms of biochemical and hematological factors.

**Conclusion:**

It seems that the treatment with shallomin syrup showed remarkable contribution to the recovery of COVID-19 induced symptoms in the patients under lopinavir/ritonavir therapy.

## Introduction

The global pandemic of Coronavirus Disease 2019 (COVID-19), caused by Severe Acute Respiratory Syndrome Coronavirus-2 (SARS-CoV-2) resulted in various complicated challenges for people and health services worldwide. So far, many clinical trials have been conducted to find effective, specific, and safe treatments for COVID-19 patients, including antiviral drugs (i.e. lopinavir/ritonavir, remdesivir, favipiravir, umifenovir, ribavirin, chloroquine, and hydroxychloroquine), antibiotics (Consortium, 2021), immune modulators, convalescent plasma (Pouladzadeh et al., 2021), interleukin inhibitors, corticosteroids, baricitinib, anakinra (Kineret^®^), tocilizumab, and anticoagulants (Teimury and Khaledi, 2020; Pouladzadeh et al., 2021). Some of these therapeutic options have shown partially good effects on COVID-19 patients’ recovery, while the lack of sufficient evidence to support their definitive effect and reports on the side effects related to these treatments limited their usage (Sahebnasagh et al., 2020; Türsen et al., 2020). Despite the potentially protective effects of widespread vaccination against different variants of COVID-19 (Byambasuren et al., 2023), it is important to acknowledge the presence of undesirable side effects (Aye et al., 2023), further research is still needed to discover effective treatments with minimal side effects (Li et al., 2023). 

For a long time, various compounds of shallot extract have been used in treating numerous types of infectious diseases, especially viral ones. Based on the available literature, shallot extract has shown meaningful antibacterial, antifungal, antioxidant, anti-*Helicobacter pylori* effects, and antiseptic properties (Mohammadi et al., 2015; Sun et al., 2019; Shahrajabian et al., 2020). Viruses like human cytomegalovirus, influenza B, parainfluenza virus type 3, herpes simplex virus types 1 and 2, and human rhino virus 2 are sensitive to shallot sap (Mikaili et al., 2013). The antiviral property of shallomin has been confirmed in the literature (Pipelzadeh et al., 2014; Shahali et al., 2019; Amin et al., 2022). Previously, the effects of shallomin in the treatment of herpes and genital warts have been reported with an excellent success (Amin and Kapadnis, 2005; Amin et al., 2012). The fraction demonstrated noticeable heat resistance and could be activated in a wide pH range (Amin et al., 2009). 

Considering the inherent unpredictability of future waves of the pandemic and the potential limitations in the long-term effectiveness of existing COVID-19 vaccines and drugs, it remains imperative to emphasize the significance of treatments targeting the symptoms induced by SARS-CoV-2 infection. Therefore, concerted endeavors to advance the development of efficacious therapeutic interventions for combating this viral infection are warranted (Yuan et al., 2023). The current study employed a randomized clinical trial design to investigate the outcomes from combined use of standard lopinavir/ritonavir therapy and the well-known shallomin extract (produced by IranAmin®) as a novel therapeutic approach for COVID-19. The examined primary outcomes included the length of hospital stay, cough, headache, muscle or joint pain, sore throat, shortness of breath, chest pain, and gastrointestinal disorders. Additionally, the study evaluated various clinical indices as the secondary outcomes, including biochemical and hematological factors, to elucidate potential side effects induced by the administration of shallomin syrup in the treated patient sample. Preliminary findings suggest that the initial use of shallomin extract (produced as co IranAmin^®^) exhibits both safety and efficacy as a complementary regimen for treating COVID-19 patients. These promising results warrant further mechanistic confirmations and subsequent clinical trial phases, potentially leading to the integration of shallomin extract in routine treatment protocol in the future.

## Materials and Methods

**Study design**

This randomized, multicenter, double-blind, parallel-group, and controlled trial study was approved by the Ethics Committee of Ahvaz Jundishapur University of Medical Sciences, Ahvaz, Iran (Ethical code: IR.AJUMS.REC.1398.995), and was registered in the national registry of clinical trials (Registration code: IRCT20200504047295N1). The trial was conducted from September 5th, 2020, to November 5th, 2020, at Razi Hospital and Sina Hospital in Ahwaz, as well as Ganjavian Hospital in Dezful, Iran. This trial included a 4-week run-in period, one-week treatment period and a 3-week follow-up period. The original total sample size was estimated according to a previous report by Cao *et al. *(Cao et al., 2020) based on Fleiss’ formula with continuity correction (Fleiss et al., 1980; Lee, 2020), as below: 

$N_{\mathrm{Fleiss}}=\frac{{\left[Z_{\frac{\alpha }{2}}\sqrt[{2}]{\left(r+1\right)p\left(1-p\right)}+Z_{\beta }\sqrt[{2}]{{\mathrm{rp}}_{0}\left(1-p_{0}\right)+p_{1}\left(1-p_{1}\right)}\right]}^{2}}{r{\left(p_{0}-p_{1}\right)}^{2}}$

Values considered in the formula were the probability of type I error (α=0.025), probability of type II error (β=0.3), proportion of disease population 1 (P0=0.5), proportion of disease population 2 (P1=0.25), Ratio of population 2 to population 1 (r=1). N_Fleiss _was calculated 57 individuals for each group. 

All protocols were developed by the principal investigators. The local institutional ethics committee of each study and clinical center oversaw the proceedings and documentation.

**Inclusion/exclusion criteria and participants blinding**

Inclusion criteria were COVID-19 infection confirmed by RT-PCR (Shanghai ZJ Bio-Tec or Sansure Biotech), and not receiving any medicines other than the approved treatment protocol, i.e. lopinavir/ritonavir therapy. Male and non-pregnant female patients being 18 years of age or older were eligible if they had a diagnostic specimen that was positive on RT-PCR, had pneumonia confirmed by chest imaging, and had an oxygen saturation (SaO_2_) of 93-96% while they were breathing ambient air or a ratio of the partial pressure of oxygen (PaO_2_) to the fraction of inspired oxygen (FiO_2_) (PaO_2_:FiO_2_) at or below 300 mg Hg (Cao et al., 2020). Exclusion criteria encompassed multiple factors, such as physician judgment against trial participation, conditions hindering safe protocol adherence, known lopinavir/ritonavir allergy or hypersensitivity, severe liver disease, contraindicated medication usage, or inability to swallow lopinavir/ritonavir (Cao et al., 2020). Furthermore, co-infection with other pathogens, severe illness, inability to consume shallomin syrup, hypersensitivity to garlic or shallomin syrup, incomplete clinical documents, or premature hospital discharge without medical authorization was considered exclusion criteria.

Shallomin oral syrup and its corresponding placebo counterpart were prepared, formulated, and labeled with unique serial numbers under the supervision of a pharmacist. Following this process, the prepared samples were administered to volunteer patients for treatment. Throughout the clinical trial, only the pharmacist possessed knowledge of the serial numbers assigned to the prepared samples. Participant enrollment, blinded treatment administration, and data collection were conducted under the supervision of physicians at each clinical site. None of the participants, providers, research supervisors, or outcome assessors were aware of the specific formulations used for treatment. Subsequently, after data collection from the clinical centers, the group treated with the formulated shallomin syrup and the group treated with the placebo samples were differentiated based on the indicated serial numbers. The obtained data were then processed under the supervision of the study designer, pharmacist, and data analysts.

**Preparation and characterization of shallomin as oral syrup**

Shallomin was prepared as reported before by Amin et al. (Amin et al., 2009) from Persian shallot, *Allium hirtifolium *that was registered by A2116401020BP code in Medicinal Plant Research Center, Ahvaz Jundishapur University of Medical Sciences, Ahvaz, Iran. Freeze-dried solid phase of the shallomin extract was dissolved as 0.1% in 0.5% ethanol (Merck, Germany) and 5% glycerol concentrations for preparation of the syrup. The extracted and formulated syrup shallomin is confirmed and registered by Iran Food and Drug Administration as co IranAmin^®^ with the registered code of 4367958851681814. At the end, these prepared final products were stored at 4ºC. 

**Treatment of the patients by the oral syrup Shallomin**

During the current study, 207 confirmed COVID-19 infected patients consenting to participate in the clinical trial were selected for treatment with the shallomin syrup during a seven-day period. In the first step and after exclusion of patients not meeting the criteria to be involved in the clinical trial, patients were divided into two groups using block randomization. Then, 64 confirmed patients were included in the control group, and the remaining 61 were in the intervention group. The control group received only the routine and confirmed national treatment protocol without additional intervention, and the intervention group received 10 ml of shallomin every 6 hours for 1 week as the complementary regimen to the confirmed treatment protocol. At the time of this clinical trial, the nationally-confirmed treatment of COVID-19 patients was lopinavir/ritonavir as Kaletra tablet (400/100 mg, respectively), taken twice a day for 14 days, besides standard COVID-19 care (Cao et al., 2020). 

**Outcomes measurement**

Before beginning the clinical trial, serum and blood samples and nasopharyngeal smears were collected from all patients for laboratory tests and RT-qPCR, respectively. The demographic data (age, gender, and main symptoms of COVID-19), COVID-19 clinical manifestations (temperature, respiratory rate, and heart rate), length of in-hospital stay, cough, headache, muscle or joint pain, sore throat, shortness of breath, chest pain, and gastrointestinal disorders were recorded as primary outcomes of COVID-19. In addition, hematological and biochemical factors, including complete blood count (CBC), C-reactive protein (CRP), erythrocyte sedimentation rate (ESR), D-dimer, prothrombin time test (PT), partial thromboplastin time (PTT), creatinine, creatinine phosphokinase (CPK), lactate dehydrogenase (LDH), low-density lipoprotein (LDL), and blood urea nitrogen (BUN) were recorded as secondary outcomes in two steps, i.e. on admission day (Pre-treatment) and on the 14^th^ day after the first treatment (Post-treatment). 

**Statistical analysis**

The normality of variables was checked by the Kolmogorov-Smirnov test. Categorical and continuous variables are presented as frequency or percentages and mean±SD (standard deviation), respectively. The chi-square test was used to compare the categorical variables, while the continuous variables were compared through an independent t-test and/or paired t-test. Analysis of covariance (ANCOVA) was used to evaluate the interaction effects of the categorical variables on the continuous dependent variables as well as to control the probable effects of other continuous variables. A p-value <0.05 was considered a definition of significance. SPSS software version 26 (IBM SPSS Inc., Chicago, IL) was used for statistical analyses. 

## Results

**Patients’ baseline data**

**Figure 1:**
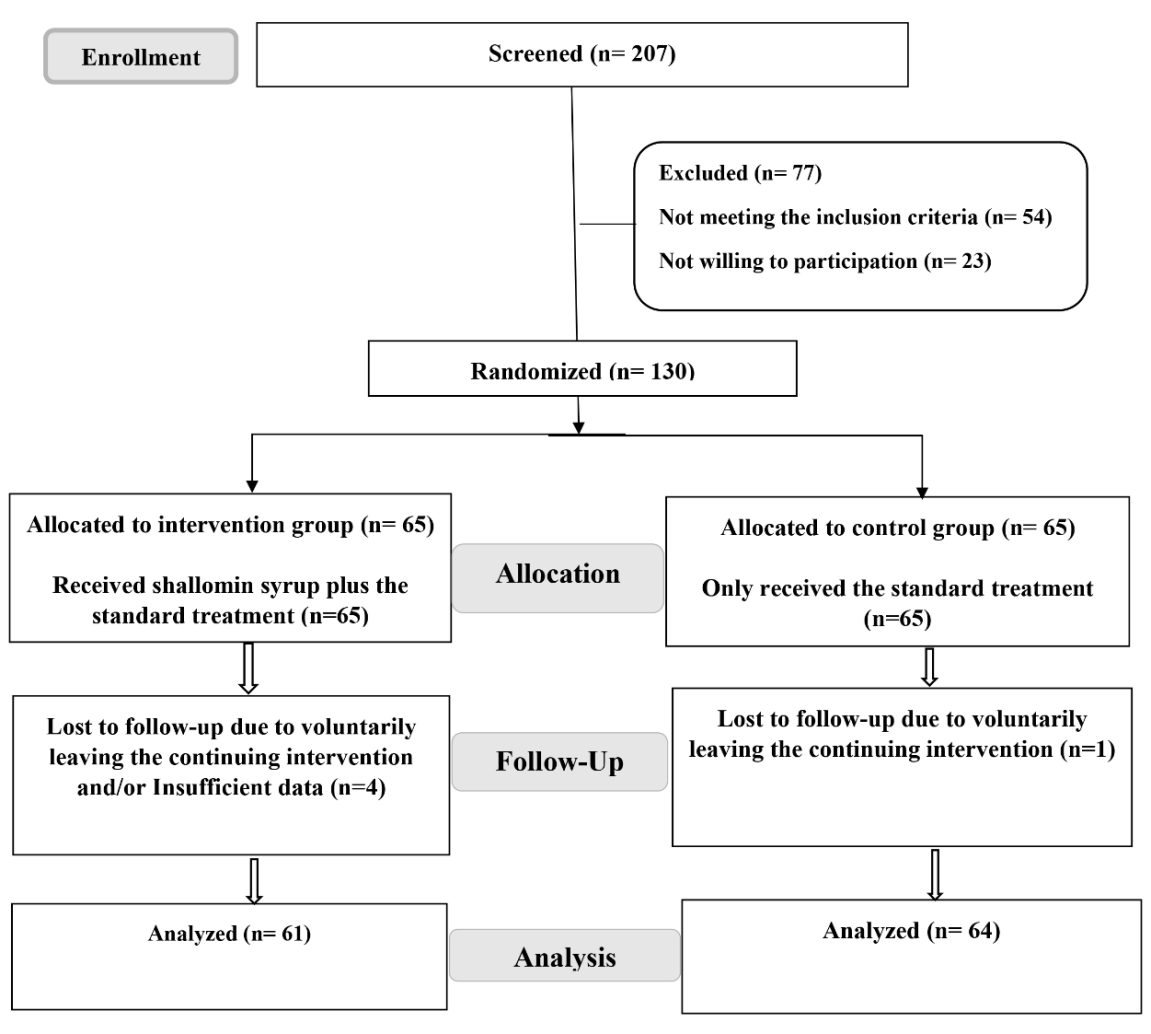
Flow diagram of recruiting the patients, group allocation, and analyzed procedures.

During the current study, 207 patients at stage 3 (hospitalized and requiring conventional oxygen) who had referred to the three clinical centers were assessed for eligibility to participate in the research. Out of this initial population, 130 patients were successfully enrolled. However, five patients (four from the intervention arm and one from the control arm) were subsequently excluded from the study due to voluntary discontinuation of the intervention and/or insufficient data. Ultimately, a total of 61 cases from the intervention arm and 64 cases from the control arm were followed up and included in the final analysis (Figure 1). 

The mean age of the cases was 55.39 years old; 57.60% were male and 42.40% were female. The mean age of the intervention group was 52.05 and that of the control group was 58.58 years old. Basic diseases (such atherosclerosis related disease or diabetes) were found among 20.30% and 34.90% of intervention and control groups, respectively. No significant difference was found between the two groups in terms of gender, sputum production, headache, muscle or joint pain, sore throat, shortness of breath, chest pain or gastrointestinal symptoms resulting from SARS-CoV-2 infection. Cough was more prevalent among the controls (98.40%) *vs* the intervention group (88.10%). See Table 1 for full baseline analysis. 

**Patients’ clinical outcomes**

We used the oral syrup of shallot bulbs, shallomin, for co-treatment of COVID-19 patients in a regimen complementary to lopinavir/ritonavir as the nationwide standard treatment. Based on our obtained data, the complementary regimen of shallomin in addition to lopinavir/ritonavir significantly reduced the recovery time of cough, myalgia, sore throat, and shortness of breath in COVID-19 patients (Table 2). 

**Table 1:** Baseline demographic and clinical characteristics for the control group (treated by lopinavir/ritonavir regimen) and the intervention group (treated by lopinavir/ritonavir plus shallomin syrup regimen). A p≤0.05 was considered significant

**Variables**	**Total (n= 125)**	**Intervention group** **[n=61 (48.80%)]**	**Control group** **[n=64 (51.20%)]**	**p-value**
Age (years old)	55.39±16.65	52.05±18.20	58.58±14.46	0.03*^T^
Gender Male: Female:	72 (57.60%) 53 (42.40%)	35 (57.40%) 26 (42.60%)	37 (57.80%) 27 (42.20%)	1
Basic disease	34 (27.2%)	12 (20.30%)	22 (34.90%)	0.10
Atherosclerosis related disease	14 (41.17%)	7 (50%)	7 (50%)	0.5
Diabetes	20 (58.82%)	9 (45%)	11 (55%)	0.14
Symptoms				
Cough	114 (93.40%)	52 (88.10%)	62 (98.40%)	0.029*
Sputum production	3 (2.50%)	1 (1.70%)	2 (3.20%)	1
Headache	53 (43.40%)	25 (42.40%)	28 (44.40%)	0.85
Muscle or joint pain	40 (32.80%)	15 (25.40%)	25 (39.70%)	0.12
Sore throat	17 (13.90%)	8 (13.60%)	9 (14.30%)	1
Shortness of breath	104 (85.20%)	48 (81.40%)	56 (88.90%)	0.31
Chest pain	35 (28.70%)	16 (27.10%)	19 (30.20%)	0.84
Gastrointestinal Symptoms	40 (32.80%)	17 (28.80%)	23 (36.50%)	0.44

^T^ Independent Samples T-test. chi-squared test

**Table 2:** Comparison of length of in-hospital stay and the recovery time of clinical signs between the intervention and control groups

**Variables**	**Recovery time of clinical signs (day)**		**p-value ** ^ҭ^
	**Intervention group [n=61]**	**Control group [n=64]**	
LOS	7.53±5.07	10.97±6.98	0.001
Cough	3.71±3.59	6.25±5.46	0.008
Headache	3.86±3.76	3.83±2.12	0.97
Muscle or joint pain	4.95±3.26	7.91±5.73	0.049
Sore throat	4.33±3.83	14.67±5.68	0.004
Shortness of breath	4.20±3.49	6.69±5	0.007
Chest pain	4.21±3.68	6.79±6.69	0.22
Gastrointestinal disorders	3.77±1.59	6.53±5.07	0.07

^ҭ^ Independent samples t-test. LOS: Length of in-hospital stay

**Patients’ vital laboratory and clinical manifestations**

During administration of the shallomin syrup in the intervention group in the present study, possible side effects or intolerance to the drug compared to the group receiving conventional treatment were also examined. However, no side effects or drug intolerance were observed in the shallomin-treated group (Table 3). Based on the intragroup analysis, the mean levels of vital signs, i.e. temperature (°C), respiratory rate, and heart rate, significantly decreased post treatment compared to before treatment in both groups (p <0.001) in a similar manner but without significance. Furthermore, between-group analyses showed no statistically significant differences out of the normal ranges at the endpoint levels of neither vital nor lab markers (p>0.05). 

**Table 3:** Comparison of the vital and laboratory markers between the intervention and control groups

**Variables**	**Intervention group** **[n=61 (48.80%)]**	**Control group** **[n=64 (51.20%)]**	**p-value (2)**
**Temperature (°C)**			
Pre-treatment	37.43±0.53	37.41±0.45	0.82
Post-treatment	36.87±0.28	36.82±1.02	0.96
Difference	-0.60±0.45	-0.68±1.05	0.61
p-value (1)	0.0001	0.0004	
**Respiratory rate**			
Pre-treatment	22_24	22_24	0.53
Post-treatment	20_20.50	20_22	0.50
Difference	-2.20	-2.28	0.80
p-value (1)	0.0001	0.0001	
**Heart rate (bpm)**			
Pre-treatment	83_89.50	83_90	0.91
Post-treatment	80_85.25	78_86	0.96
Difference	-8_ -2.75	-9 _ -3	0.34
p-value (1)	0.0002	0.0001	
**WBC per microliter**			
Pre-treatment	8245.54±3957.55	7433.22±3203.45	0.22
Post-treatment	9353.02±5261.73	9110.64±4342.51	0.81
Difference	996.65±5033.12	1878.30±3590.24	0.33
p-value (1)	0.19	0.0008	
**Lymphocyte (per mm3)**			
Pre-treatment	1097.54±879.30	1114.70±662.35	0.90
Post-treatment	1665.01±1489.19	1460.14±1347.82	0.50
Difference	495.74±1420.54	328.64±1369.97	0.57
p-value (1)	0.03	0.10	
**Neutrophils (per mm3)**			
Pre-treatment	7021.03±3621.42	5711.70±2827.32	0.04
Post-treatment	7551.41±4625.88	6962.70±3851.88	0.51
Difference	192.11±3002.70	1461.46±3115.83	0.05
p-value (1)	0.68	0.002	
**Platelets (per mm3)**			
Pre-treatment	249.89±107.08	253.64±108.90	0.85
Post-treatment	294.56±124.14	251.76±103.76	0.22
Difference	40.38±117.33	-8.52±108.50	0.04
p-value (1)	0.15	0.58	
**CRP (mg/L)**			
Pre-treatment	73±57.95	68.67±62.77	0.85
Post-treatment	40.02±52.68	23.31±25.54	0.22
Difference	-37.62±80.54	-41.76±59.59	0.83
p-value (1)	0.03	0.0007	
**ESR (mm/hr)**			
Pre-treatment	49±27.66	57.47±26.98	0.10
Post-treatment	37.15±26.43	43.98±24.88	0.22
Difference	-11.56±28.60	-16.75±26.47	0.40
p-value (1)	0.01	0.0001	
**D-dimer (ng/ml)**			
Pre-treatment	49±27.66	57.47±26.98	0.10
Post-treatment	37.15±26.43	43.98±24.88	0.22
Difference	-11.56±28.60	-16.75±26.47	0.20
p-value (1)	0.01	0.0001	
**PT (s) (INR)**			
Pre-treatment	12.55±1.27	12.67±1	0.60
Post-treatment	12.79±1.63	12.49±0.884	0.30
Difference	0.40±1.36	-0.18±1.47	0.06
p-value (1)	0.04	0.52	
**PTT (s)**			
Pre-treatment	33.11±7.37	31.33±5.25	0.16
Post-treatment	30.45±6.96	31.42±5.87	0.50
Difference	-1.77±10.27	-0.30±4.90	0.41
p-value (1)	0.29	0.68	
**Creatinine levels (mg/dl)**			
Pre-treatment	0.95±0.24	1.10±1.02	0.05
Post-treatment	0.89±0.23	0.50±0.62	0.20
Difference	-0.077±0.162	-0.073±0.316	0.93
p-value (1)	0.004	0.11	
**CPK (U/L)**			
Pre-treatment	171.85±178.91	154.45±149.72	0.64
Post-treatment	87.46±82.87	90.07±64.85	0.89
Difference	-68.61±200.45	-43.57±83.60	0.54
p-value (1)	0.09	0.01	
**LDH (Unit/L)**			
Pre-treatment	694.40±319.66	706.39±370.95	0.85
Post-treatment	588.32±229.32	651±345.68	0.33
Difference	-112.77±351.39	-116±277.64	0.96
p-value (1)	0.04	0.009	
**LDL (Unit/L)**			
Pre-treatment	97.47±98.96	85.22±33.85	0.50
Post-treatment	82.23±38.51	83.19±36.31	0.92
Difference	3.88±37.87	-1.67±13.53	0.44
p-value (1)	0.60	0.50	
**BUN (mg/dl)**			
Pre-treatment	18.52±8	18.70±8.10	0.50
Post-treatment	21.20±11.06	20.88±7.66	0.92
Difference	3.42±7.53	2.51±8.44	0.59
p-value (1)	0.60	0.50	

BUN, Blood urea nitrogen; CPK, Creatine phosphokinase; LDH, Lactate dehydrogenase; LDL, Low-density lipoprotein

## Discussion

According to the most recent update of the NIH COVID-19 Treatment Guidelines (April 20, 2023), the severity of the disease is categorized into five stages as follows: being hospitalized for reasons other than COVID-19, hospitalization without the need for oxygen supplementation, hospitalization requiring conventional oxygen therapy, hospitalization requiring high-flow nasal cannula (HFNC) oxygen or non-invasive ventilation (NIV), and hospitalization requiring mechanical ventilation (MV) or extracorporeal membrane oxygenation (ECMO) (https://www.covid19treatmentguidelines.nih.gov/tables/management-of-hospitalized-adults-summary/). As previously mentioned, the twice-daily oral administration of lopinavir/ritonavir (400 mg/100 mg) was among the approved treatment protocols for COVID-19 patients in 2020 (Cao et al., 2020). However, a clinical trial conducted by Alexander M. Kaizer *et al.* in 2023 demonstrated that this regimen did not result in significant improvements in symptom resolution or reduction of hospitalization individuals with COVID-19 (Kaizer et al., 2023). In addition, Kandeel and colleagues conducted a study establishing that the treatment regimen involving lopinavir/ritonavir was not effective as a standalone therapy or in combination with interferon for COVID-19 patients (Kandeel et al., 2023). Presently, there are three recommended therapeutic protocols for hospitalized COVID-19 patients who require conventional oxygen. These protocols involve the use of remdesivir alone, a combination of dexamethasone and remdesivir (or dexamethasone alone if remdesivir is unavailable), and the administration of oral baricitinib or intravenous tocilizumab (Collins et al., 2023). 

The antiviral properties of shallot have been established previously (Taran et al., 2006). Mnayer et al. (2014) found that shallot extracts had considerable antimicrobial and antioxidant effects (Mnayer et al., 2014). Although there are conflicting outcomes observed with many proposed treatments for COVID-19 (Focosi et al., 2022; Vahedian-Azimi et al., 2022), the complementary treatment of shallomin has demonstrated significant effects on the duration of hospitalization for COVID-19 patients. Reducing the time of hospitalization for COVID-19 patients is one of the main aims in effective treatment of the patients. A study by Baud et al. (2020) showed that average hospitalization time for confirmed COVID-19 patients (from the onset of symptoms that need intensive care) was about 10 days (Baud et al., 2020). One of the consequential results in the present study was the effect of the complementary treatment on the length of hospitalization of COVID-19 patients. While in the intervention group, the average number of hospitalization days was 7.53±5.07, this value was 10.97±6.98 for the control group, clearly showing the significant effect of the complementary treatment on decreasing the duration of admission in hospital and its positive effect on patients' recovery process. 

Based on the report by Rong-Hui Du et al. at 2020 in a prospective cohort study, mortality rate of hospitalized COVID-19 patients due to multiple organ failure, especially respiratory failure and heart failure, was 11.7%, and the mean±SD of duration from admission to death was 13.7±8.3 days (range 3-33 days) (Du et al., 2020). The mortality rate in the patients of the present study was 4 (6.55%) people in the intervention group and 11 (17.18%) in the control group, indicating the meaningful reduction in death events. Therefore, remarkable shortening of hospitalization and reduction of mortality in the intervention patients may be the most important clinical outcomes of treating the patients with the oral syrup shallomin as a complementary medical regimen. It seems that the small study population may be the main reason for the non-significant difference in death reduction observed. Although we used shallomin as a complementary treatment simultaneously with oral lopinavir/ritonavir (400 mg/100 mg), ineffectiveness of the oral lopinavir/ritonavir treatment in hospitalized and non-hospitalized COVID-19 patients was confirmed and wildly accepted at the time of this paper’s publication (Kandeel et al., 2023). Therefore, the reported beneficial effects observed in this study can be attributed exclusively to the treatment with shallomin syrup.

During the current study, we could not find any noticeable side effect of shallomin, while many of proposed protocols that are used for treatment of COVID-19 patients could cause serious side effects, such as the increased risk of bradycardia (Beyls et al., 2020), nausea, and breathing problems (Goldman et al., 2020), development of anemia (Praveen et al., 2020), the immunosuppression (King et al., 2020; Russell et al., 2020), and increased alanine aminotransferase during the treatment (Alattar et al., 2020).

Emerging of new variants of SARS-CoV-2 with new attributes may also question the efficacy of approved vaccines and medicines. In such situation, complementary regimen medicines may present a potential to effectively cure or at least decrease the SARS-CoV-2 induced symptoms. In the current randomized, double-blinded and multicenter trial, we investigated the efficacy and safety of an ethylene acetate fraction of Persian shallot named shallomin with specific anti SARS-CoV-2 effects as an oral syrup for patients infected by SARS-CoV-2 in combination with lopinavir/ritonavir treatment. Based on the obtained results, treatment with shallomin was significantly associated with a shorter length of in-hospital stay, a shorter length of the recovery time for myalgia, and faster cure of sore throat and shortness of breath. Furthermore, this indicated co-treatment of COVID-19 patients could also decrease the reported mortality rate. Based on the assessment of hematological and inflammatory markers, no side effects were observed in the intervention group when compared to the control group. However, it is important to acknowledge the limitations of this study, as is the case with other investigations examining the efficacy of vaccines or medications against COVID-19. These limitations include the potential impact of new variants of SARS-CoV-2, variations in symptom presentation depending on the susceptibility and underlying conditions of infected individuals, variations in therapeutic protocols across different clinical centers, and variations in protocols endorsed by international health organizations.
